# The diversity and dynamics of tumor-associated macrophages in recurrent glioblastoma

**DOI:** 10.3389/fimmu.2023.1238233

**Published:** 2023-09-04

**Authors:** Lingyun Zhang, Yu Jiang, Gao Zhang, Shiyou Wei

**Affiliations:** ^1^ Institute of Thoracic Oncology and Department of Thoracic Surgery, West China Hospital, Sichuan University, Chengdu, China; ^2^ School of Biomedical Sciences, The Chinese University of Hong Kong, Hong Kong, Hong Kong SAR, China; ^3^ Department of Neurosurgery, West China Hospital, Sichuan University, Chengdu, China; ^4^ Faculty of Dentistry, The University of Hong Kong, Sai Ying Pun, Hong Kong, Hong Kong SAR, China

**Keywords:** recurrent glioblastoma, tumor microenvironment, immunotherapy, microglia, monocyte-derived macrophages, single-cell

## Abstract

Despite tremendous efforts to exploit effective therapeutic strategies, most glioblastoma (GBM) inevitably relapse and become resistant to therapies, including radiotherapy and immunotherapy. The tumor microenvironment (TME) of recurrent GBM (rGBM) is highly immunosuppressive, dominated by tumor-associated macrophages (TAMs). TAMs consist of tissue-resident microglia and monocyte-derived macrophages (MDMs), which are essential for favoring tumor growth, invasion, angiogenesis, immune suppression, and therapeutic resistance; however, restricted by the absence of potent methods, the heterogeneity and plasticity of TAMs in rGBM remain incompletely investigated. Recent application of single-cell technologies, such as single-cell RNA-sequencing has enabled us to decipher the unforeseen diversity and dynamics of TAMs and to identify new subsets of TAMs which regulate anti-tumor immunity. Here, we first review hallmarks of the TME, progress and challenges of immunotherapy, and the biology of TAMs in the context of rGBM, including their origins, categories, and functions. Next, from a single-cell perspective, we highlight recent findings regarding the distinctions between tissue-resident microglia and MDMs, the identification and characterization of specific TAM subsets, and the dynamic alterations of TAMs during tumor progression and treatment. Last, we briefly discuss the potential of TAM-targeted strategies for combination immunotherapy in rGBM. We anticipate the comprehensive understanding of the diversity and dynamics of TAMs in rGBM will shed light on further improvement of immunotherapeutic efficacy in rGBM.

## 1 Introduction

Glioblastoma (GBM) is the most prevalent and malignant type of brain tumors. Nearly 90% of GBM relapse despite the standard of care involving surgery, radiotherapy, and chemotherapy (1). Recurrent GBM (rGBM) generally differs from primary GBM in their molecular and histological characteristics, intra-tumor heterogeneity, immune microenvironment, and biological behaviors due to the therapeutic pressure and clonal selection, which contributes to the aggressiveness and therapeutic resistance of rGBM (2). Therefore, patients with GBM rapidly succumb to this disease, with a median overall survival of 12-15 months after initial diagnosis and a 5-year survival rate of less than 10% (3, 4); however, due to the lack of abundant high-quality rGBM samples, most current studies focus on primary GBM, while the biology of rGBM remains largely unknown, and practical therapeutic approaches against rGBM are lacking. Therefore, it is instrumental to understand the biology of rGBM for developing effective therapeutic strategies and improving the clinical outcome of patients with GBM.

Recently, emerging immunotherapies, including immune checkpoint blockade (ICB), vaccine, and chimeric antigen receptor (CAR) T-cell therapy, have revolutionized the therapeutic landscape of multiple types of cancers (5); however, several clinical trials have revealed disappointing therapeutic efficacy of immunotherapy in rGBM, and the underlying mechanisms remain incompletely elucidated (6–8). The tumor microenvironment (TME) is pivotal in orchestrating immune activity and modulating response to immunotherapy. GBM is a typically “cold tumor” with an immunosuppressive TME featured by the paucity of cytotoxic T cells (CTLs) and the abundance of immunosuppressive cells such as myeloid cells (8–11). Tumor-associated macrophages (TAMs) are the most predominant non-malignant cells infiltrating GBM, which consist of tissue-resident microglia and monocyte-derived macrophages (MDMs). Growing evidence has suggested pro-tumor functions of TAMs in GBM include aggravating tumor growth and metastasis, angiogenesis, immunosuppression, treatment resistance, *etc* (12–14). Besides, the level of TAMs is markedly increased in rGBM, which in turn is associated with poor prognosis of patients (8, 15).

TAMs are incredibly plastic and heterogeneous, exhibiting diverse phenotypes and functions when responding to the environment-specific stimuli. Recently, high-resolution methodologies [e.g., single-cell RNA-sequencing (scRNA-seq)] have been instrumental in identifying and characterizing various subsets of TAMs with distinct functions in GBM (13, 16). Interestingly, emerging evidence has revealed the dynamic alterations of TAMs during disease progression and therapeutic resistance in rGBM. Meanwhile, different TAM-targeted therapeutic approaches have been developed, showing promising potential in multiple types of cancers, including rGBM (17, 18). Therefore, an elaborated understanding of the complexity of TAMs and molecular mechanisms underlying the tumor-promoting roles of TAMs in rGBM is vital to facilitating TAM-modulating treatment in order to overcome resistance of rGBM to immunotherapy.

In this review, we first provide a concise overview of characteristics of TME and immunotherapy for patients with rGBM. We then describe the biology of TAMs in rGBM, including their origins, categories and functions. In particular, we review recent advances regarding the phenotypic and functional diversity of TAMs in rGBM at the single-cell resolution, and focus on distinctions between tissue-resident microglia and MDMs, the characterization of specific subsets, and the dynamic changes of TAMs during tumor evolution and treatment in GBM. Finally, we highlight the potential of therapeutically targeting TAM as the basis for combination immunotherapy for patients with rGBM.

## 2 TME and immunotherapy in rGBM

### 2.1 TME of rGBM

In the context of cancer, various types of immune cells enter the central nervous system (CNS) by disrupting the blood-brain barrier. The TME of GBM is dominated by immunosuppressive cells, including TAMs, myeloid-derived suppressor cells (MDSC), and regulatory T cells (Treg) (9, 10). Besides, genetic alterations are associated with the immune status of GBM, and diverse immune landscapes in four molecular subtypes of GBM [neural, pro-neural (PN), classical (CL), and mesenchymal (MES)] have been documented (19, 20). For example, tumor-infiltrating CTLs were scarce in the CL subtype but abundant in the MES subtype (21). Also, a preponderance of TAMs was identified in the MES subtype (22). In addition, the *IDH-1* mutation, which frequently occurrs in the PN subtype, is correlated with reduced Tregs and monocyte signatures, PD-L1 expression, and a favorable prognosis (23–25). Thus, the heterogeneity of TME that is associated with cancer genetics provides a foundation for tailoring therapies for patients with GBM.

Crucially, the TME altered by the treatment results in a unique TME for rGBM that differs from that of primary GBM. For instance, 82% of rGBM lost the expression of *epidermal growth factor receptor variant III* (*EGFRvIII*). *EGFRvIII* is an immunogenic mutation widely detected and constitutively activated in primary GBM, indicating that immunologic escape occured after a period of progression-free survival in rGBM (26). Recently, it was reported that CD103^+^ Tregs with upregulated lipid metabolism accumulated in response to ICB therapy and concurrent radiotherapy, which hindered the cytotoxic activity of CTLs in GBM (27). Additionally, rGBM exhibited an increase in the infiltration of CD68^+^ macrophages following anti-angiogenic therapy, suggestive of the potential role of TAMs in controlling therapeutic resistance and tumor relapse (15). More dynamic changes of TAMs during tumor progression and treatment will be reviewed in the following sessions. Taken together, the highly heterogeneous, dynamic and immunosuppressive TME is a key player contributing to anti-tumor immune evasion in rGBM.

### 2.2 Progress and challenges of immunotherapy in rGBM

ICB therapy could inhibit the immune checkpoint pathways such as programmed death-1/programmed death-ligand 1 (PD-1/PD-L1) signaling, thus alleviating T cell exhaustion and enhancing CTLs-mediated tumor killing (28). Despite the therapeutic success of anti-PD-1/PD-L1 treatment in multiple types of cancers, the phase III clinical trial Checkmate 143 reported that anti-PD-1 antibody nivolumab failed to achieve survival benefits compared with bevacizumab in rGBM patients (6, 29–31). In contrast, another clinical trial conducted by Cloughesy et al. demonstrated that OS in rGBM patients treated with neoadjuvant anti-PD-1 therapy (surgery following pembrolizumab) was improved compared with adjuvant-only treatment, which was accompanied by increases in the expression levels of genes related to T cells and interferon (IFN)-γ within the tumor (32). Besides, several studies have suggested the promising anti-tumor effects of ICB-based combination therapy in pre-clinical GBM mouse models, but clinical trials are needed to determine the clinical efficacy (33–36). Overall, current evidence hints that a single ICB treatment might be insufficient to revert the immunosuppressive TME of rGBM and elicit satisfactory efficacy. Therefore, it is worth investigating an ICB-based combination treatment against rGBM.

The tumor-specific peptide vaccination provides a promising approach to trigger specific immune responses by targeting tumor-associated antigens (TAAs). rGBM possesses a broad spectrum of TAAs, including CD133, gp100, EGFRvIII, IL-13Rα2, Wilms’ tumor 1 (WT1), HER2, *etc* (37–40). Several clinical trials have demonstrated the survival benefit of GBM-specific peptide vaccination, but the therapeutic response was hampered by pre-treatment lymphopenia, which highlighted the necessity of more rigorous selection criteria for patient enrollment (41). On the other hand, given the crucial role of dendritic cells (DCs) in antigen presentation and activation of CTLs, DC vaccination therapy has also exhibited an encouraging effect in treating patients with rGBM (42–45); however, it is incredibly time-consuming to isolate and purify autologous DCs, making it challenging to exploit DCs-based immunotherapy for rapidly progressing rGBM.

Recently, CAR-T cell immunotherapy has presented an attractive anti-tumor method and succeeded in treatment of hematological malignancies (46–48). Several studies have demonstrated the safety and feasibility of IL-13Rα2-specific and HER2-specific CAR T cells in patients with rGBM (49–52); however, researchers reported the limited efficacy of EGFRvIII-specific CAR-T cell therapy in patients with rGBM (7). EGFRvIII was highly expressed in primary GBM but exhibited a specific loss or decreased expression in tumors resected after CAR-T cell therapy (7, 53, 54). Apart from EGFRvIII antigen escape, the adaptive immunosuppressive response was observed in the TME upon CAR-T therapy, suggested by the upregulated expression of inhibitory molecules, including PD-L1, TGF-β, IDO, and IL-10 and infiltration of Tregs (7). Currently, the durable clinical efficacy of CAT-T cell therapy in rGBM is hindered by the short lifespan of CAR-T cells, the poor infiltration of T cells in tumor tissues, tumor heterogeneity, and antigen escape, which needs to be addressed in the future (55).

Despite the recent breakthrough of immunotherapy in a subset of patients with rGBM, there are still many obstacles in the practical application. More efforts should be made to solve the issues regarding the optimum approach, treatment timing, patient selection, and combination modalities to augment the efficacy of immunotherapies for patients with rGBM.

## 3 Origin, classification, and roles of TAMs in rGBM

### 3.1 Origin and recruitment of TAMs in rGBM

Microglia are the brain-resident macrophages originating from yolk sac-derived embryogenetic precursors. Under normal physiological conditions, microglia comprise 10% of the adult brain cell populations, represent the main component of brain macrophages, and play an essential role in maintaining the immune homeostasis of CNS (16, 56). Upon inflammatory stimulation, such as infection and cancer, bone marrow-derived monocytes in the peripheral blood are recruited to the tumor site and then differentiate into macrophages. Various recruitment signals have been recognized, including colony-stimulating factor-1 (CSF-1), monocyte chemoattractant protein-1 (MCP‐1), and stromal-derived factor (SDF)-1α derived from tumor cells and other cells in the TME (12, 57–60).

The term TAMs in GBM include both tissue-resident microglia and MDMs. It is challenging to distinguish or separate microglia from MDMs using conventional approaches (e.g., flow cytometry) due to the lack of specific markers (61); however, growing evidence has demonstrated the dramatic distinctions in preferential localizations and functions between these two subpopulations. For example, Chen et al. found that MDMs accounted for most TAMs in GBM and were mainly located in perivascular areas. Inversely, microglia only represented a minor TAM population, usually appearing in the peritumoral zones (58). Moreover, microglia-derived TAMs are predominant in primary GBM but are outnumbered by MDMs following recurrence, especially under hypoxia (62). Phenotypically, MDMs upregulate immunosuppressive cytokines and show an altered metabolism compared to microglial TAMs (63). More studies are needed to dissect the exact origin and specific roles of TAM populations in GBM.

### 3.2 Classification of TAMs in rGBM

Based on the polarization status and regulatory functions under inflammation, macrophages are divided into classically activated macrophages (M1, pro-inflammatory) and alternatively activated macrophages (M2, anti-inflammatory) (64). M1 macrophages can be induced by lipopolysaccharide (LPS), IFN-γ, granulocyte–macrophage colony-stimulating factor (GM-CSF) or Toll-like receptor signaling pathway. M1 macrophages spur inflammation by releasing cytokines such as IL-1α, IL-1β, IL-6, IL-12, and tumor necrosis factor (TNF)-α. On the other hand, M2 macrophages are stimulated by IL-4, IL-10, IL-13, and glucocorticoid. M2 macrophages express PD-L1 and exert immunosuppressive functions by secreting IL-10, arginase-1, TGF-β, *etc* (64, 65). Generally, M1 macrophages exert an anti-tumor role, whereas M2 macrophages play a pro-tumor role. Several markers distinguish the M1 from the M2 phenotype, e.g., CD80, CD86, and MHC-II for M1, CD163 and CD206 for M2, although they are not absolutely specific (66, 67). CSF-1, TGF-β1, macrophage inhibitory cytokine 1 (MIC-1), osteopontin (OPN), and Periostin produced by GBM cells recruit and polarize macrophages to a tumor-supporting M2-like phenotype (68–71). CD163 and CD206 are highly expressed in perivascular macrophages in the brain tumor cores and are associated with an immunosuppressive TME (72).

M1/M2 nomenclature is proposed mainly based on *in vitro* data when macrophages were stimulated with type 1 or 2 cytokines. This nomenclature remains oversimplified, albeit widely used (66). Indeed, macrophages are highly plastic and heterogeneous, with the capacity of being reprogrammed into distinct phenotypes by different microenvironmental stimuli. Besides, canonical M1 and M2 markers are co-expressed in individual cells, implying that macrophages could possess a mixed M1/M2 phenotype (63). Beyond M1/M2, the more complicated phenotypic and functional diversity of TAMs in GBM has been recently appreciated (73–75). Next, we will review the diverse roles of TAMs in regulating progression of GBM, and summarize recent advances that reveal the complexity of TAMs in GBM based on single-cell omics approaches.

### 3.3 Functions of TAMs in GBM

**Figure 1:**
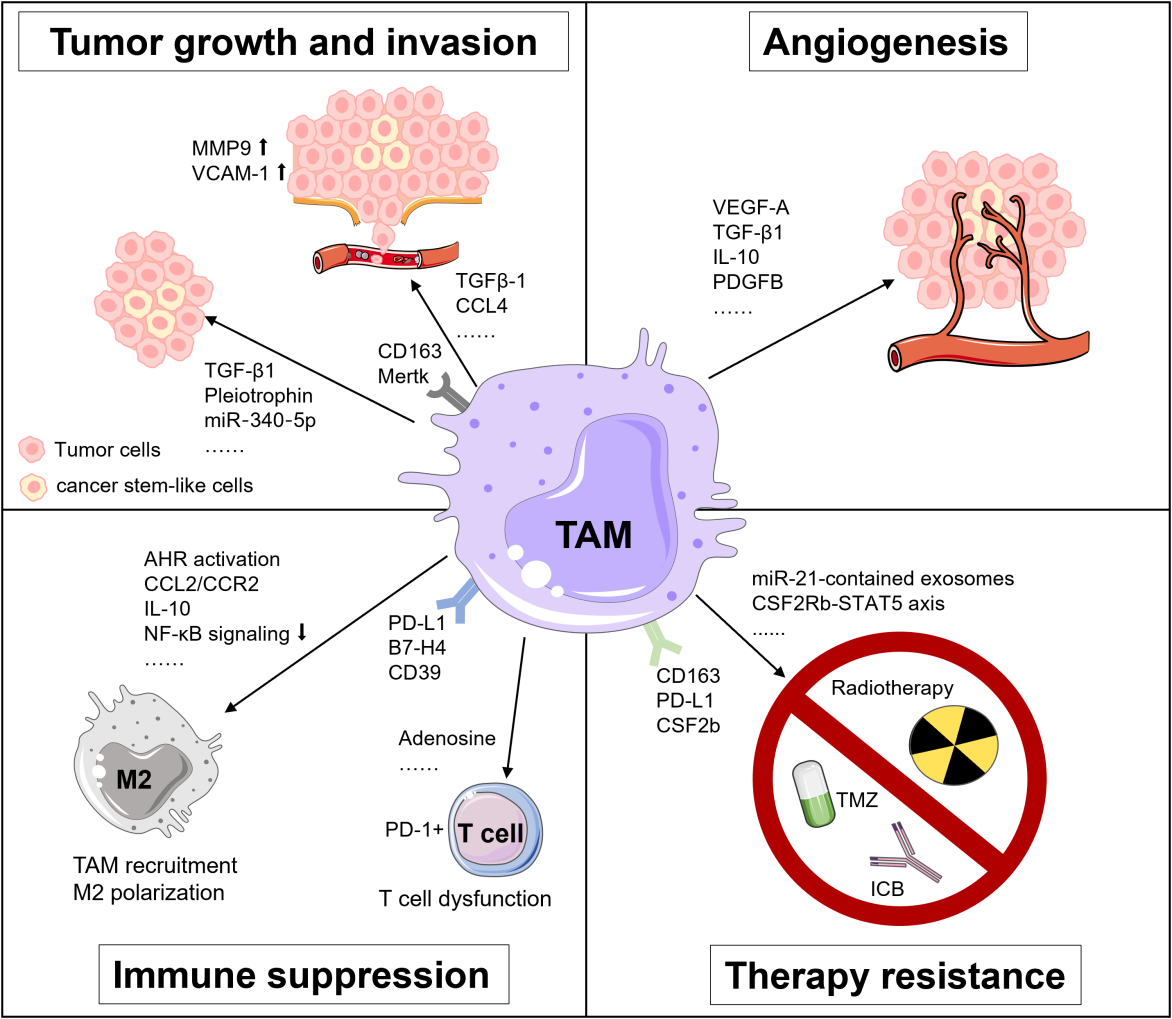
Tumor-supportive functions of TAMs in GBM. TAMs foster tumor growth and invasion, angiogenesis, immunosuppression and treatment resistance in GBM via multiple pathways.

TAMs are involved in tumor development and progression via releasing various factors and interacting with other cells in multiple malignancies (17, 76). In GBM, the pro-tumor roles of TAMs are well documented that implicate the importance of TAMs as a therapeutic vulnerability in GBM, involving tumor growth, invasion, angiogenesis, immunosuppression, and treatment resistance (**Figure 1**) (12, 18).

#### 3.3.1 TAMs aggravate tumor growth and invasion

The molecular interaction between TAMs and tumor cells is critical for regulating tumor growth and invasion. For instance, TAMs secrete TGF-β1 to recruit CD133^+^ cancer stem-like cells (CSCs) (77). Pleiotrophin (PTN) derived from CD163^+^ M2 macrophages binds to its receptor protein tyrosine phosphatase receptor type Z1 (PTPRZ1) on the surface of CSCs. The binding of PTN and PTPRZ1 contributes to the stemness maintenance and tumorigenic capacity of CSCs, thus accelerating the growth of GBM (78). Besides, TAMs upregulate the expression of metalloproteinase 9 (MMP-9) of CSCs via TGF-β1 and CCL4-CCR5 signaling to enhance the GBM invasiveness (79). Also, the vascular cell adhesion molecule-1 (VCAM-1)-mediated interaction between macrophages and GBM cells reinforces GBM invasion (80). Additionally, Liu et al. unveiled that a miR‐340‐5p‐macrophage feedback loop regulated tumor progression and was related to a poor prognosis for patients with GBM (81). Moreover, TAMs expressing myeloid-epithelial-reproductive tyrosine kinase (MerTK), a critical tyrosine kinase for phagocytosis function, are associated with tumor growth (82).

#### 3.3.2 TAMs promote angiogenesis

The rapid proliferation of tumor cells accelerates the consumption of oxygen and nutrients in the TME, rendering a highly hypoxic environment for GBM, especially in its core region (83). Under the hypoxic condition, TAMs produce angiogenesis-promoting cytokines, chemokines, and growth factors, like vascular endothelial growth factor A (VEGF-A), a well-known factor for vascularization and immunosuppression in multiple cancers, including GBM (83–85). Besides, Cui et al. found that GBM-induced M2-like macrophages secreted more TGF-β1 and IL-10. These anti-inflammatory cytokines facilitated endothelial capillary proliferation and angiogenic sprouting through integrin (αvβ3) receptors and Src-PI3K-YAP signaling. Hence, dual blockade of integrin (αvβ3) and cytokine receptor (TGFβ-R1) could suppress the neovascularization of GBM induced by the TAM-endothelial interaction (86). Moreover, a recent study by Zhu et al. suggested that the expression of cat eye syndrome critical region protein 1(CECR1*)* was upregulated in M2 macrophages and correlated with microvascular density in GBM. Mechanistically, CECR1 mediated the crosstalk between macrophages and vascular mural cells via the PDGFB-PDGFRβ signaling axis, leading to recruitment of pericytes, migration, and tumor angiogenesis (87). Collectively, TAMs exert potent pro-angiogenic properties in GBM, implying that therapeutically targeting TAMs may present an attractive way against rGBM.

#### 3.3.3 TAMs orchestrate immune suppression

The highly immunosuppressive TME represents a hallmark of GBM, which is primarily attributed to TAMs via multiple mechanisms. For instance, decreased IKBKB expression and NF-κB signaling in TAMs support M2 polarization and correlate with defective expression of immune/inflammatory genes, resulting in immune suppression in GBM (88). Accordingly, NF-κB-targeted therapy could reverse M2 polarization, induce tumor regression and improve survival of a GBM mouse model in T cell-dependent manner (89). Besides, Takenaka and colleagues recently uncovered mechanisms by which TME controlled TAMs and T cells in GBM. Kynurenine produced by GBM cells elicited the activation of aryl hydrocarbon receptor (AHR) in TAMs, which further increased the expression of *CCR2* and boosted TAM recruitment via the CCL2/CCR2 axis. Aside from that, AHR drove the expression of ectonucleotidase CD39 in TAMs and led to the dysfunction of CTLs via adenosine accumulation. Moreover, elevated expression of AHR was associated with glioma grade and unfavorable prognosis in patients with GBM (90).

Immune checkpoint molecules are critical inducers of immunosuppressive TME. Reportedly, GBM could upregulate PD-L1 expression in circulating monocytes and TAMs through the IL-10 signaling axis in an autocrine/paracrine manner. *In vitro*, macrophages stimulated by IL-10 induced T cell apoptosis, which could be attenuated by inhibiting IL-10 and its receptor (91). Besides, Yao et al. unveiled that CD133^+^ CSCs activated the expression of B7-H4 in TAMs via the IL6/JAK/STAT3 pathway. Such B7-H4-mediated crosstalk between glioma-initiating cells and TAMs was associated with a bleak prognosis of human GBM (92).

#### 3.3.4 TAMs mediate therapeutic resistance

TAMs are involved in therapuetic resistance of GBM to temozolomide (TMZ), radiotherapy, and immunotherapy. For example, TAMs release oncomiR-21-contained exosomes, which upregulate the production of PDCD4, SOX2, STAT3, IL-6, and TGF-1 in GBM cells, rendering resistance of GBM to TMZ. Pacritinib, a STAT3 inhibitor, could overcome resistance to TMZ by decreasing miR-21-enriched exosomes from TAMs (93). Besides, Miyazaki et al. revealed that TMZ-resistant GBM cells produced M2-related cytokines including IL-10, IL-4, IL-13, and CSF-1, and PD-L1 expression. Upon *in vivo* anti-PD-L1 antibody administration, TMZ-resistant GBM tumor tissues showed abundant infiltration of CD163^+^ M2 macrophages. Expectedly, remarkable anti-tumor efficacy was achieved using a combination therapy of anti-PD-L1 antibody plus IPI-549, a PI3Kγ inhibitor that could skew M2 macrophages to M1 macrophages (94). Moreover, dynamic transcriptional alterations of TAMs in the irradiated and recurrent tumors have been observed in mouse and human GBM, and CSF-1R inhibition could overcome resistance of pre-clinical models to radiotherapy (95).

As for the significance of TAMs in mediating resistance of GBM to immunotherapy, Simonds et al. previously discovered the association between PD-L1^+^ TAMs and resistance to ICB by comparing the TME of human ICB-refractory GBM and ICB-responsive tumors using cytometry by time-of-flight (CyTOF) (96). Additionally, through integrated analyses of multi-dimensional data, Lee et al. demonstrated that neoadjuvant anti-PD-1 blockade induced conventional type 1 DC (cDC1) and activation of T cells but failed to eliminate immunosuppressive TAMs in rGBM (97). Interestingly, in the mouse model of brain metastases, pro-inflammatory activation of TAM which was mediated by the compensatory CSF2Rb–STAT5 signaling axis fostered tumor recurrence after CSF1R inhibition. Furthermore, blockade of CSF1R combined with STAT5 signaling inhibitor could sustain tumor control and rectify adaptive resistance to CSF1R inhibition (98). All of these findings highlight the potential benefit of TAM-targeted therapeutic intervention for overcoming treatment resistance in rGBM and metastatic brain tumors.

## 4 Emerging diversity of TAMs in GBM at the single-cell resolution

As mentioned before, owing to the plastic and heterogenous nature of TAMs, the linear M1/M2 activation theory is insufficient to explain the *in vivo* complexity of TAMs in GBM (66). On the other hand, despite the well-documented anti-tumor functions of M1 macrophages and pro-tumoral functions of M2 macrophages, the prognostic value of CD163^+^ and CD206^+^ M2 macrophages was controversial in different cohorts of patients with GBM, emphasizing an unmet need to decipher the exact function of specific TAM subtypes in GBM (20, 99); however, limited by conventional approaches, hurdles exist to distinguish and characterize TAM subpopulations in GBM. In the past years, the application of high-dimensional and high-resolution techniques has enabled us to decipher unprecedented macrophage subclusters in the brain under homeostasis and disease, and moved us beyond the binary M1/M2 polarization paradigm. Herein, we will review recent advances in the phenotypic and functional diversity of TAMs in GBM and provide insights into the therapeutic potential of TAM-based strategies for patients with rGBM.

### 4.1 Distinctions between tissue-resident and monocyte-derived macroohages in GBM

**Figure 2:**
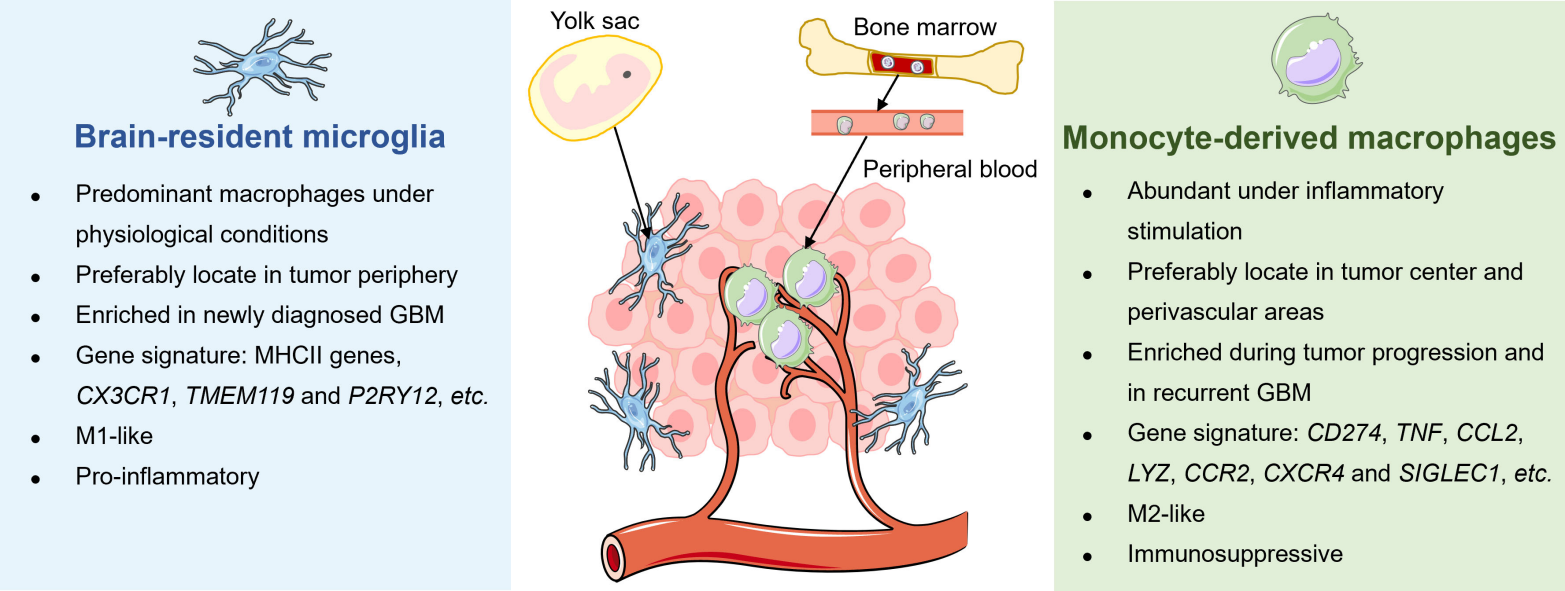
Distinctions between tissue-resident microglia and monocyte-derived macrophages in GBM. The tissue-resident microglia originate from the yolk sac and represent the primary macrophages in the brain under physiological conditions to maintain immune homeostasis. Under malignant conditions, microglia preferably locate in the periphery of newly diagnosed GBM, exhibiting a pro-inflammatory M1-like phenotype. Meanwhile, bone marrow-derived monocytes in circulation are recruited into the brain and differentiate into macrophages. Unlike microglia, MDMs are enriched during tumor progression, particularly in tumor core and perivascular regions of rGBM, and present immunosuppressive M2-like properties.

Microglia and MDMs are different in the spatial distribution, enrichment extent, phenotypic and functional characteristics during disease progression in GBM (**Figure 2**). A study by Darmanis et al. suggested that the majority of myeloid cells within the tumor center preferentially exhibited gene signatures of macrophages, whereas microglia-related genes were mainly expressed in myeloid cells located in the surrounding space in human GBM (100). Similarly, by analyzing RNA-seq data from the human GBM cohort, Kim et al. recently confirmed that microglial genes (*CX3CR1*, *TMEM119*, and *P2RY12*) were mainly expressed in the periphery, while activated macrophage genes (*TNF*, *CCL2*, *LYZ*, *CCR2*, *CXCR4*, and *SIGLEC1*) were predominantly detected within the core tumor regions (74). Besides, Muller’s group has reported that differing from microglia, MDMs were usually enriched in perivascular and necrotic areas in human glioma (63).

Several studies have attempted to dissect longitudinal changes of TAM composition throughout tumor evolution and recurrence in GBM. For instance, Yeo et al. reported a progression of TME from M1-like proinflammatory microglia towards an M2-like pro-tumorigenic infiltrating macrophages during tumor growth in the mouse model of GBM. Notably, a similar transition was observed in tumor biopsies derived from patients with low-grade glioma and GBM (101). Besides, Pombo Antunes et al. profiled myeloid cells in mouse and human GBM at new diagnosis and recurrence by employing scRNA-seq and cellular indexing of transcriptomes and epitopes by sequencing (CITE-seq) technical platforms. The researchers found that microglia-derived TAMs were predominant in initial tumors but were outnumbered by MDMs in recurrent tumors, especially under hypoxic conditions. Although microglia and MDMs exhibited functional specialization to some extent, both of them showed a convergent angiogenic and T-cell suppressive capacity (62).

Emerging evidence has suggested additional phenotypic and functional differences between microglia and MDMs. By performing scRNA-seq of *IDH*-mutant human gliomas, Venteicher et al. unmasked a continuous rather than a bimodal distribution of transcriptional signatures of microglia/macrophages, underscoring the plasticity of cellular states of TAMs. Besides, the macrophage signature, but not the microglia signature, was associated with clinical grade and increased vascularity in gliomas (102). Additionally, Ochocka et al. identified distinct transcriptional programs of microglia and monocytes/macrophages in mice bearing GBM via scRNA-seq analysis. The transcriptional responses of macrophages were associated with the activation of immunosuppressive genes such as *Cd274* encoding PD-L1, while microglia had higher expression levels of major histocompatibility complex II (MHC-II) genes in a sex-specific manner (103). Consistently, Muller et al. determined that MDMs tended to express immunosuppressive cytokines and to undergo metabolic reprogramming compared with microglia in human gliomas (63). Collectively, these results shed light on the dynamic alterations of TAMs with different origins in response to disease status and microenvironment stimulation in GBM.

### 4.2 Identification and characterization of specific subsets of TAMs in GBM

Substantial efforts have been made to identify specific subsets of TAMs with the most promising therapeutic potential in GBM (**Table 1**). For instance, by integrating analyses of scRNA-seq and CyTOF data, Sankowski et al. mapped microglial states in the human brain under healthy and malignant conditions, and uncovered a disease-associated transcriptional signature in TAMs from patients with GBM. These TAMs exhibited down-regulation of the microglia core signature and concomitant up-regulation of inflammatory, metabolic, and hypoxia-related genes, including *SPP1*, and several type I interferon genes, *APOE*, and *CD163*. Furthermore, the top differentially expressed proteins, including HLA-DR, TREM2, APOE, CD163, and GPR56, could be detected in a TAM subset via CyTOF, providing a possibility for therapeutically targeting specific TAM states in GBM (75).

**Table 1:** Specific TAM subsets in GBM identified through single-cell omics technologies.

Species	Marker/Signature	Function/enriched pathway	Technique	Dataset
Human	*SPP1*, *HLA-DR*, *TREM2*, *APOE*, *CD163*, *GPR56*	↓: Microglia core signature. ↑: Inflammatory, metabolic and hypoxia-associated molecules.	scRNA-seq and CyTOF	GSE135437 (75)
Human	CD73^+^	Immunosuppression. Modulate macrophage polarization and T cell infiltration.	scRNA-seq and CyTOF	PRJNA588461 (104)
Human	S100A4^+^	Impede immune response, including phagocytic activity of macrophages, production of IFN-γ and activation of T cells, thus favoring glioma growth	scRNA-seq	GSE182109 (105)
Human	MACRO^+^	Anti-inflammatory. Loss of pro-inflammatory pathways (interferon response, allograft rejection, TNFα signaling via NFKB) and antigen presentation.	scRNA-seq	GSE141383 (106)
Human	↑: *CX3CR1*, *NLRP1*, *IL1B*, *APOE*, *PDGFRA*, *SOX2*. ↓: *P2RY12*, *TMEM119.*	Pro-inflammatory and proliferative. Promoting tumor progression through IL-1β secretion.	scRNA-seq	PRJNA669369 (107)
Human	HMOX1^+^	Mediate T cell dysfunction via IL-10 release.	scRNA-seq and spatial transcriptomics	https://osf.io/4q32e/ (108)
Human	CD14^+^ERO1A^+^	Hypoxia-response signatures. Associated with tumor angiogenesis, invasion, and poor prognosis	scRNA-seq	GSE135045 (109)
Human	MPO^+^	Less interactions with endothelial cells, enhanced cytotoxic functions.	Imaging mass cytometry	Available upon request (73)
Mouse	CD169^+^	Pro-inflammatory and anti-tumor. Induce T cells and NK cells infiltration.	scRNA-seq	GSE201559 and GSE200533 (74)

↑ Upregulation; ↓ Downregulation.

Using similar approaches, Goswami et al. determined the persistence of a unique population of CD73^+^ macrophages in patients with GBM upon anti-PD-1 treatment. They further demonstrated the critical function of CD73^+^ macrophages in conferring resistance to ICB, which was mediated by the modulation of macrophage polarization and T cell infiltration. Compared with wild-type mice, the authors detected a decrease of immunosuppressive CD206^+^Arg1^+^VISTA^+^PD-1^+^CD115^+^ myeloid cluster and an increase of iNOS^+^ myeloid cells in CD73-deficient mice. Upon the treatment of CD73-knockout mice with the combination with anti-CTLA-4 plus anti-PD-1, the tumor burden was significantly alleviated and survival was improved, along with the elevated ratio of the granzyme B^+^ effector CD8 T cells to the CD206^+^ macrophages in the TME. The study indicated that CD73 was a promising immunotherapeutic target to augment anti-tumor immune responses to the combination immunotherapy in GBM (104).

Recently, through multi-regional and -dimensional analyses at the single-cell level, Abdelfattah et al. sought to discover immune modulatory targets in GBM. scRNA-seq analysis identified that *S100A4* was highly expressed in innate and adaptive immunosuppressive cells, including Tregs, exhausted T cells, and three subsets of pro-tumorigenic myeloid cells. The immunofluorescence staining confirmed the expression of S100A4 in immunosuppressive CD163^+^, CD206^+^ macrophages, and FOXP3^+^ T cells in human glioma. Moreover, a higher expression of S100A4 was markedly associated with a worse survival and was recognized as an independent prognostic indicator for patients with GBM. Subsequent animal experiments and functional analyses demonstrated the roles of S100A4 in impeding immune response, including phagocytic activity of macrophages, production of IFN-γ and activation of T cells, thus favoring the growth of glioma, supporting S100A4 as a promising immunotherapeutic target (105).

Additionally, Chen et al. integrated analyses of newly generated and published single-cell RNA-seq data, which identified a tumor-supportive subcluster of TAMs characterized by the scavenger receptor MARCO, almost exclusively expressed in *IDH1*-wild-type (*IDH*-WT) GBM. Moreover, MACRO was reportedly detrimental in melanoma and non-small cell lung cancer. The expression of *MARCO* in bulk tumors was also associated with a disappointing prognosis and mesenchymal subtype in the GBM cohort. Further analysis observed the loss of pro-inflammatory pathways (interferon response, allograft rejection, and TNFα signaling via NFKB) and antigen presentation in MACRO^+^ TAMs, supporting its anti-inflammatory phenotype. Altogether, the study revealed a novel TAM subpopulation driving the progression of GBM and implied a potential strategy for MACRO^+^ TAM-targeted therapy (106).

Increasing evidence has also elucidated how TAMs orchestrate the immunosuppressive TME through various crosstalk with their neighboring components. Through scRNA-seq analysis of human GBM, Liu et al. identified a unique pro-inflammatory and proliferative subpopulation of microglia, marked by upregulated expression of *CX3CR1, NLRP1, IL1B, APOE, PDGFRA*, and *SOX2*. The microglia were activated by TGF-β1 derived from *SETD2*-mut/*IDH*-WT tumor cells, and accelerated tumor progression via secreting IL-1β. Notably, depletion of TGF-β1/TGF-β RI successfully reduced the pro-inflammatory and proliferative microglia and restrained tumor growth (107). Through integrative analysis of single-cell and spatial transcriptomics data of human GBM, Ravi et al. revealed that a subset of HMOX1^+^ microglia and macrophages released IL-10 and mediated T cell dysfunction, thus fostering an immunosuppressive TME (108). Additionally, a specific CD14^+^ERO1A^+^ TAM cluster with detrimental prognostic value in human primary GBM has been identified, which showed a gene signature enriched in hypoxia-response, invasion and extracellular matrix organization. The CD14^+^ERO1A^+^ TAM cluster, together with two hypoxia-dependent MES-like tumor cells expressed VEGFA, indicating their contribution to the induction of angiogenesis in GBM via interacting with endothelial cells (109).

Contrary to the well-known tumor-supportive functions of TAMs, studies also showed that several subsets of macrophages favor anti-tumor immunity against GBM. For example, through the analysis of scRNA-seq data, Kim et al. unraveled that CD169^+^ TAMs were IFN-responsive macrophages, which produced pro-inflammatory chemokines, hence inducing the infiltration of T cells and NK cells in human and mouse gliomas. Mechanistically, CD169^+^ TAMs originated from CCR2^+^ blood monocytes, and IFN-γ derived from NK cells was critical for recruiting CD169^+^ macrophages into gliomas. CD169 boosted the phagocytosis capacity of macrophages through ligands of apoptotic tumor cells and ignited antigen-specific T cell responses. Moreover, the clearance of CD169^+^ TAMs impaired anti-tumor responses mediated by T cells and shortened the survival of mice bearing glioma (74). Recently, Karimi et al. characterized the immune landscape of primary and metastatic human brain tumors at the single-cell level by applying imaging mass cytometry. Specifically, they identified a unique subpopulation of myeloperoxidase (MPO)-positive neutrophil-like macrophages, which was related to reduced interactions with endothelial cells, enhanced cytotoxic functions and survival benefit for patients with GBM (73).

The remarkably distinct and even inverse roles of TAMs in orchestrating GBM progression reflect their plasticity and heterogeneity. Therefore, it is necessary to identify specific targets and mechanisms for tailoring TAMs-modulating therapy regimens. Still, the vast diversity of TAMs in GBM remains incompletely illustrated, highlighting an urgent demand for more investigations that utilize high-dimension and high-resolution approaches and platforms.

### 4.3 The dynamic alterations of TAMs during disease progression and treatment

Several studies have investigated alterations of TAMs during tumor evolution in GBM at the single-cell level (**Table 2**). For instance, Rajendran et al. not only revealed an immune-activated feature displayed by TAM clusters in low-grade murine glioma but also demonstrated an immunosuppressive property in murine high-grade murine glioma, accompanied by the restriction of T cell trafficking and activation. They further identified high expression of CD74 and its binding partner, macrophage migration inhibition factor (MIF) in distinct TAM populations, which was subsequently validated in human samples and supported the CD74-MIF axis as a potential target for TAMs (11). As mentioned above, the preponderance of TAMs underwent a transition from M1-like proinflammatory microglia to M2-like pro-tumorigenic macrophages during GBM progression, which was conserved in human and mouse. The transition was concurrent with a disruption of the blood-brain barrier and an explosive growth of malignant cells (101).

**Table 2:** The dynamic alterations of TAMs during disease progression and treatment in GBM at the single-cell resolution.

Species	Therapy	Alterations of TAMs	Technique	Transcriptome datasets
High-grade vs. low-grade glioma				
Mouse (validated in human via IF and RNA-seq analysis)	N/A	TAM clusters displayed an immune-activated feature in low-grade glioma but adopted an immunosuppressive property in high-grade glioma, accompanied by restriction of T cell trafficking and activation.	scRNA-seq	GSE221440 (11)
During tumor growth of GBM				
Mouse (validated in human via flow cytometry)	N/A	The predominance of TAMs switched from M1-like proinflammatory microglia towards M2-like protumorigenic macrophage during GBM progression.	scRNA-seq	GSE195848 (101)
Recurrent vs. primary GBM				
Human	Radiotherapy and chemotherapy	TAM infiltration increased in MES-like rGBM and was inversely correlated with tumor purity	snRNA-seq and RNA-seq	EGAD00001009871; EGAD00001009964 (110)
Human and mouse	Surgical resection, adjuvant radiotherapy and chemotherapy	Microglia were predominant in ND tumors, but were outnumbered by MDMs following recurrence, especially in hypoxic niche. Notable genes that were enriched in recurrent versus ND TAMs were related to monocyte chemotaxis, IFN signaling and phagocytosis.	scRNA-seq and CITE-seq	EGAS00001004871 (human); GSE163120 (mouse) (62)
Human	TMZ, IR and surgical resection	Bone marrow-derived monocytic lineage cells increased and microglia reduced in all tumor-associated innate immune cells at recurrence. Although both subsets had more activated M1 and M2 cells at recurrence, most of them were remained M0 state.	snRNA, scATAC-seq, spatial transcriptomic/proteomic assays, exome-seq	EGAS00001004909 (111)
Mouse (validated in human via RNA-seq and IF staining)	Radiotherapy	The abundance of MDMs increased relative to microglia in rGBM. MDMs and microglia converged upon a common phenotype at recurrence, which is potentially regulated by SMAD and RBPJ.	RNA-seq of isolated MG and MDMs	GSE99537 (95)
Human	Surgical resection followed by radiotherapy and chemotherapy	Microglial population prominently decreased in rGBM.	scRNA-seq	HRA003075 (112)
Human	Mainly chemotherapy and radiotherapy	MDMs were enriched in rGBM, while microglia were enriched in primary GBM.	snRNA and spatial transcriptomics	GSE228500 (113)
Human	Neoadjuvant PD-1 blockade therapy	Myeloid populations sustainedly expressed T-cell-suppressive checkpoints, including TIGIT and CTLA-4, and displayed reinforced interactions between T cells upon PD-1 blockade therapy	scRNA-seq and CyTOF	GSE154795 (97)
Human	Anti-PD-1 treatment	*MACRO* expression decreased in post-treatment tumors compared with pre-treatment tumors in responders rather than non-responders in anti-PD-1-treated rGBM patients.	scRNA-seq	GSE141383 (106)

N/A, Not applicable.

Hoogstrate et al. conducted a large-scale transcriptome analysis of paired primary-recurrent GBM resections of patients following standard therapy and suggested that rGBM preferentially progressed to MES-like subtype (110). Macrophages are known to be recruited by GBM stem cells and induce the MES-like state of GBM cells (110, 114, 115). Consistently, Hoogstrate et al.’s study identified significant increase of TAM infiltration in MES-like rGBM, which was inversely correlated with tumor purity, supporting the essential role of TAMs in favoring MES-like GBM progression at recurrence (110).

As mentioned above, Pombo Antunes et al. compared the immune landscape of newly diagnosed (ND) GBM versus rGBM following surgery, adjuvant radiotherapy and chemotherapy through scRNA-seq and CITE-seq analyses. The TME of ND GBM mainly consisted of TAMs (82–97%), followed by T cells (2–20%), while rGBM displayed a more diverse immune compartment including increased T cells, NK, B cells and monocytes. Microglia formed the major TAM fraction in ND GBM, but MDMs outcompeted microglia in rGBM, especially in the hypoxic tumor niche. TAMs in recurrent versus ND GBM displayed higher expression of genes related to monocyte chemotaxis, IFN signaling, and phagocytosis (62). Additionally, a single-cell multi-omics analysis conducted by Wang et al. demonstrated an increase of bone marrow-derived monocytic lineage cells and a reduction of microglia in all tumor-associated innate immune cells at recurrence upon standard-of-care therapy including TMZ, IR and surgical resection. Although both subsets had more activated M1 and M2 macrohages at recurrence, most of them were classified as M0 state without expressing either program above. It may be attributed to the oversimplification of M1/M2 paradigm, and a continuous modal for macrophage classification is demanded (111).

In another study, by analysing RNA-seq data of isolated microglia and MDMs in pre- and post-treatment murine gliomas, Akkari and colleagues identified stage-dependent transcriptional reprogramming of these two TAM subpopulations in irradiated murine glioma. In line with previous studies, the results confirmed the increased abundance of MDMs relative to microglia in rGBM compared with primary GBM (62, 111–113). MDMs and microglia maintained their ontogeny-based identities and converged upon a common phenotype at recurrence, which is potentially regulated by SMAD and RBPJ. Notably, recurrence-specific transcriptional changes of TAMs were also observed in human rGBM. Inhibition of CSF1-R could counteract the recurrence-induced gene signature alterations in TAMs, enhance the efficacy of radiation therapy and delay tumor regrowth in pre-clinical mouse models (95). Collectively, the findings disclosed the dynamics and plasticity of individual TAM populations during radiation treatment and provided novel insight into improving the treatment landscape in GBM.

Additionally, a study by Lee et al. also mapped the landscape of infiltrating immune cells in GBM, with a particular focus on alterations in TME following neoadjuvant PD-1 blockade therapy. By exploiting high-dimensional proteomics, scRNA-seq and quantitative multiplex immunofluorescence (mIF), the authors determined increased activation and infiltration of T cells and cDC1 after ICB treatment; however, TAMs and monocytes maintained the dominance of tumor-infiltrating immune cells upon anti-PD-1 therapy. Although the interferon-mediated T-cell chemotactic factors (such as CXCL9, CXCL10, and CXCL11) were secreted in myeloid populations after PD-1 blockade, these cells sustainedly expressed T cell-suppressive checkpoints, including TIGIT and CTLA-4. Furthermore, the analysis of scRNA-seq data recognized reinforced interactions between T cells and myeloid cells through TIGIT- and CTLA-4-related signaling after PD-1 blockade therapy, which could impede optimal and durable activation of CTLs. Therefore, additional strategies targeting TIGIT and/or CTLA-4 may enhance the strength and durability of CTL-mediated anti-tumor response of GBM to immunotherapy (97).

Besides, in the above-described study, decreased expression of *MACRO* was observed in post-treatment tumors compared with pre-treatment tumors in responders rather than non-responders in a longitudinal cohort of patients with rGBM treated with anti-PD-1. However, there were no apparent changes in expression of *MARCO* after treatment in another longitudinal cohort of patients of GBM with standard therapy. These findings suggested that *MACRO* was altered upon treatment in an immunotherapy-specific and response-dependent manner (106).

More investigations are required to delineate the dynamics and plasticity of TAMs during treatment and determine the mechanisms by which TAMs modulate therapy outcomes, thus providing translational relevance for enhancing therapeutic efficacy in GBM.

## 5 Targeting TAMs for boosting immunotherapy against rGBM

The strong tumor-promoting activity of TAMs has highlighted its promising potential as a therapeutic target against rGBM. Multiple TAM-targeted approaches have been explored in preclinical and clinical settings for patients with rGBM, mainly including: i). reduction of the recruitment of TAMs into tumors; ii). elimination of TAMs within tumors; iii). reprogramming of TAMs. Since these strategies have been reviewed recently, we provide a concise summary here (13, 14, 17, 18, 116).

### 5.1 Reduction of TAM recruitment

The inhibition of TAM infiltration could be realized by directly blocking signalings between chemokines and their receptors. For example, CCL2 derived from tumor cells recruit CCR2^+^ myeloid cells; hence CCR2 antagonist could directly reduce TAM infiltration and improve the efficacy of ICB in murine GBM (117). As mentioned above, kynurenine produced by GBM cells led to AHR activation, further promoting CCR2 expression and enhancing TAM recruitment. In this case, AHR antagonist effectively suppressed GBM growth via reducing CCL2/CCR2-mediated TAM infiltration (90). Besides CCL2/CCR2 axis, other chemoattractant-receptor interactions have also shown therapeutic potential in GBM, such as lysyl oxidase (LOX)/β1 integrin, OPN/αvβ5 integrin, and slit guidance ligand 2 (SLIT2)/Roundabout 1 and 2 (ROBO1/2) (71, 118, 119).

### 5.2 Elimination of TAMs

CSF-1R is expressed on macrophages and critical for regulating the survival, proliferation, differentiation, and polarization of TAMs by binding with its ligands CSF-1 and IL-34 (120, 121). Targeting CSF-1R using antibodies or small molecule inhibitors has represent a powerful strategy to deplete TAMs and induce TAM repolarization in various types of cancers, including GBM (122–124). It is worth noting that monotherapy of targeting CSF-1R was insufficient to elicit satisfactory efficacy, and combination therapy with immunotherapy or radiation demonstrated better clinical outcome, which highlight the necessity of combination strategy in clinical exploration (125, 126). Besides, along with our extended understanding of the complexity of TME and the heterogeneity of TAM subpopulations, we should realize that the unbiased depletion of the whole TAM cluster may not be an optimal option, because it is likely to eliminate beneficial TAM subpopulations and influence other TME components. Therefore, more efforts should be made to identify specific tumor-supporting subtypes of TAMs (e.g., CD73^+^, MACRO^+^, and HMOX1^+^ TAMs) and develop targeted therapy across ravious scenarios.

### 5.3 Reprogramming of TAMs

In spite of the detrimental function of TAM subsets, macrophage play an essential role in phagocytosis and antigen presentation, which is beneficial for the activation of anti-tumor immunity (127). Therefore, rather than macrophage clearance and recruitment inhibition, another attractive strategy is to reprogram/re-deucate TAMs, i.e., reprogram immunosuppressive TAMs to immune-supportive TAMs by restoring their phagocytic and antigen presenting capacities (76). To achieve reprogramming of TAMs in GBM, multiple approaches have been developed. For instance, the blockade of phagocytosis checkpoint pairs, e.g., CD47/SIRPα, CD24/Siglec-10 could augment the phagocytic ability of TAMs (128–132). To unleash the immune-stimulatory capacity of TAMs, blockade of CSF1/CSF1R, stimulation of CD40/CD40L, as well as inhibition of PI3Kγ, IL-6, SLIT2, monoacylglycerol lipase (MAGL) have shown promising targetable potential and are worth further investigation (94, 119, 124, 133–135).

Notably, no single TAM-targeted agent has been successful in clinical trials for patients with GBM. Given the complexity of the TME and the close interplay between TAMs, tumor cells, and other non-malignant cells, combination therapy emerges as an attractive option. For instance, in a mouse model of GBM, the SDF-1α inhibitor in combination with VEGF blockade was more efficient in suppressing TAM recruitment, reducing tumor vasculature and improving survival compared with monotherapy of VEGF blockade (136). As discussed in the above, CSF1-R blockade in combination with radiotherapy substantially inhibited tumor growth and prolonged survival by reversing transcriptional changes of TAM induced by radiation in pre-clinical glioma models, thus overcoming resistance to radiotherapy (95). Similarly, a triple combination of oncolytic virus expressing IL-12, and anti-PD-1 plus anti-CTLA-4 antibodies synergistically cured pre-clinical murine GBM via increasing M1-like polarization and the ratio of effector T cells to Tregs (137). Further investigation regarding combination therapy regimens in the clinical setting is dispensable for strengthening the efficacy of immunotherapies for patients with rGBM.

## 6 Conclusions and perspectives

rGBM has been characterized by a highly immunosuppressive TME and an extremely low response to immunotherapy. TAMs, originating from microglia and peripheral monocytes, represent the dominant non-malignant cells in the TME of rGBM. TAMs exert various tumor-supportive functions, contributing to tumor growth and invasion, angiogenesis, immune evasion, and treatment resistance. More importantly, TAMs are plastic and heterogeneous, displaying more complicated phenotypes beyond the binary M1/M2 polarization. Recently, single-cell omics methodologies have enabled us to characterize the dynamics and diversity of TAMs in the TME of GBM at the single-cell resolution. Microglia and MDMs show different spatial distribution and exhibit distinctive transcriptional alterations across disease stages. Besides, specific subsets of TAMs with different functions have been determined in the context of rGBM, e.g., the pro-tumor MACRO^+^, CD73^+^, HMOX1^+^, and S100A4^+^ macrophages, and anti-tumor CD169^+^ and MPO^+^ macrophages, further underscoring the complexity of TAMs. Moreover, several studies have interrogated the dynamic changes of TAMs responding to treatment and microenvironmental stimulation, providing novel insights into how TAMs modulate therapeutic response and resistance. Therefore, harnessing TAMs via different approaches may be feasible in treating rGBM, and TAM-based combination therapy regimens have started to show a promising potential.

Still, dynamics and diversities of TAMs in the context of rGBM, and underlying molecular mechanisms remain incompletely clarified, which warrants further investigations and integrated analyses of high-dimensional and high-resolution data, such as spatial scRNA-seq, single-cell proteomics, and single-cell sequencing assay for transposase-accessible chromatin (scATAC-seq). More studies in the near future should focus on i). distinguishing microglia from MDMs; ii). identifying specific tumor-supportive and tumor-suppressive TAM subclusters; iii). delineating the stage- and therapy-specific reprogramming of TAMs in longitudinal cohorts; iv). dissecting the cellular crosstalk between TAMs and other cells; v). exploring rationale-based combination therapy modalities in clinical trials targeting TAMs. Ultimately, comprehensively understanding TAMs and their interplay with other cells will be instrumental for optimal immunomodulation and enhanced immunotherapeutic efficacy for patients with rGBM.

## Author contributions

GZ and SW conceived and designed the study. LZ wrote the original draft. YJ, GZ and SW revised the manuscript. All authors contributed to the article and approved the submitted version.
